# Supplementary material for: Differentiation between Weissella cibaria and Weissella confusa Using Machine-Learning-Combined MALDI-TOF MS
Source: Int J Mol Sci. 2023 Jul 2;24(13):11009. doi: 10.3390/ijms241311009 (PMC10341702; doi:10.3390/ijms241311009)
Supplement: Supplementary file 1 [file ijms-24-11009-s001.zip › ijms-2432717-supplementary.pdf]

Supplementary Materials

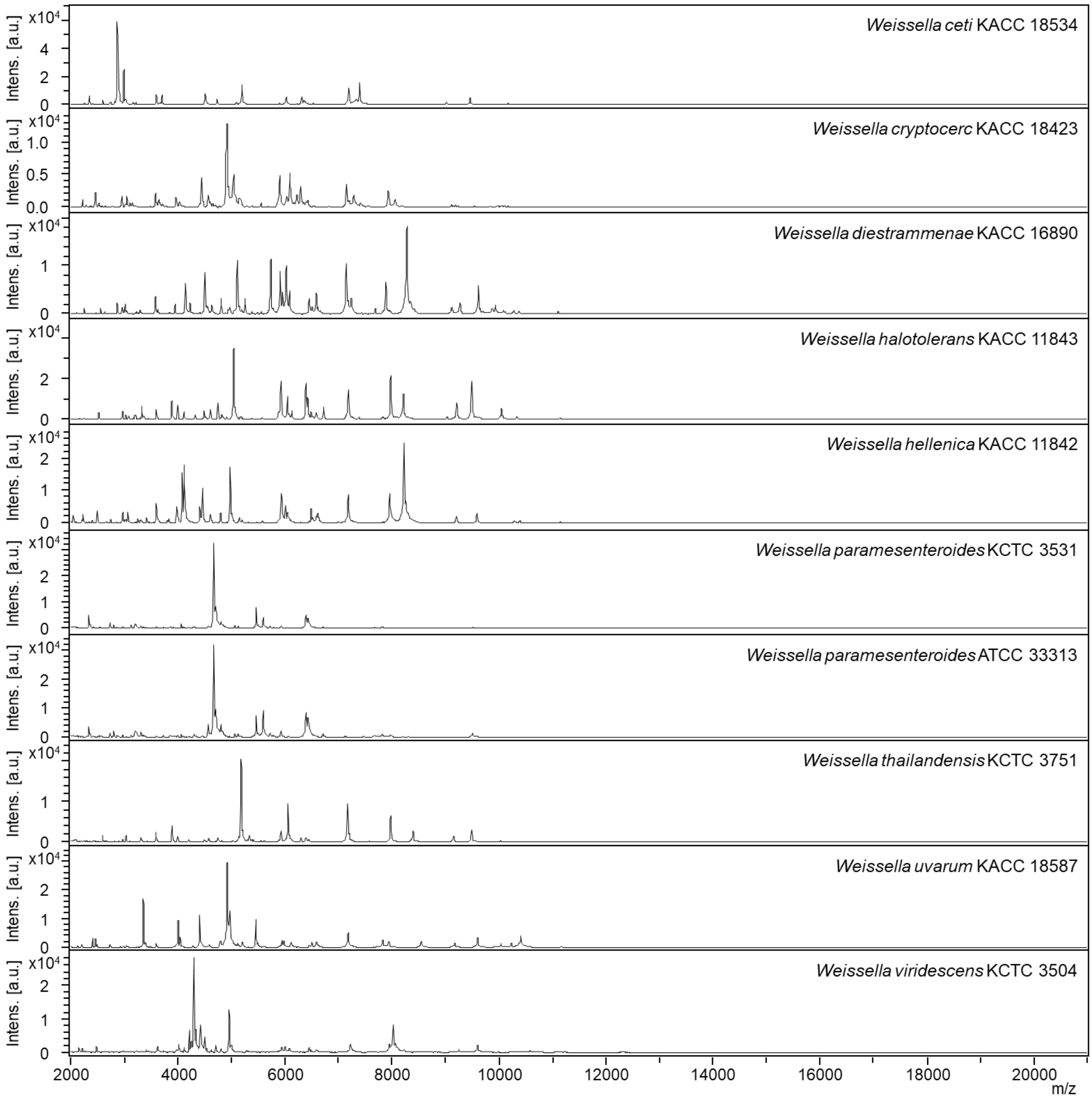

Supplementary Figure S1. Mass spectra of reference strains of non-target *Weissella* species; m/z, mass-to-charge ratio; a.u., arbitrary units.

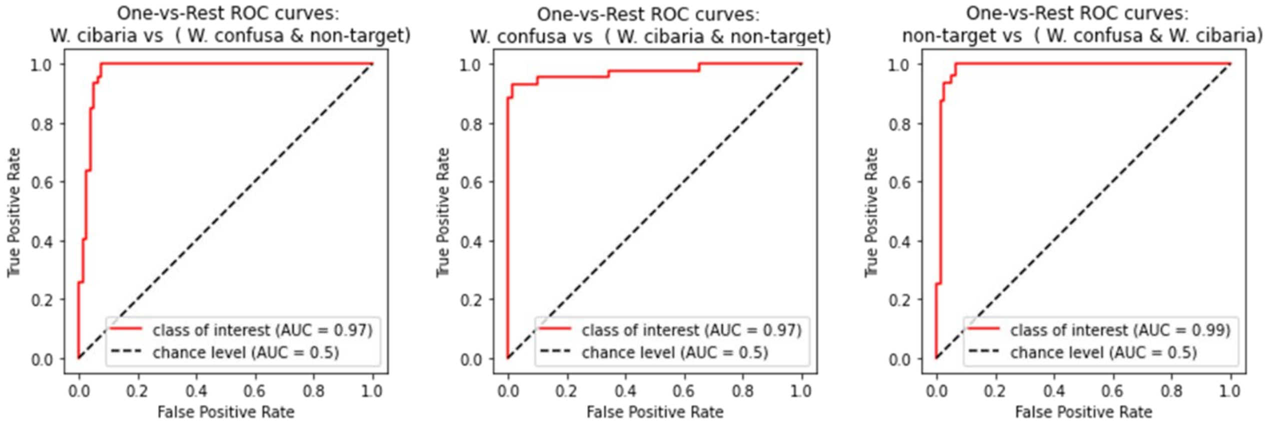

Supplementary Figure S2.

The developed ANN model was utilized to analyze the ROC curves and AUC, showing the following: (Left) The classification performance between *W. cibaria* and the other two classes, (middle) The classification performance between *W. confusa* and the other two classes, (Right) The classification performance between non-target samples and the other two classes.

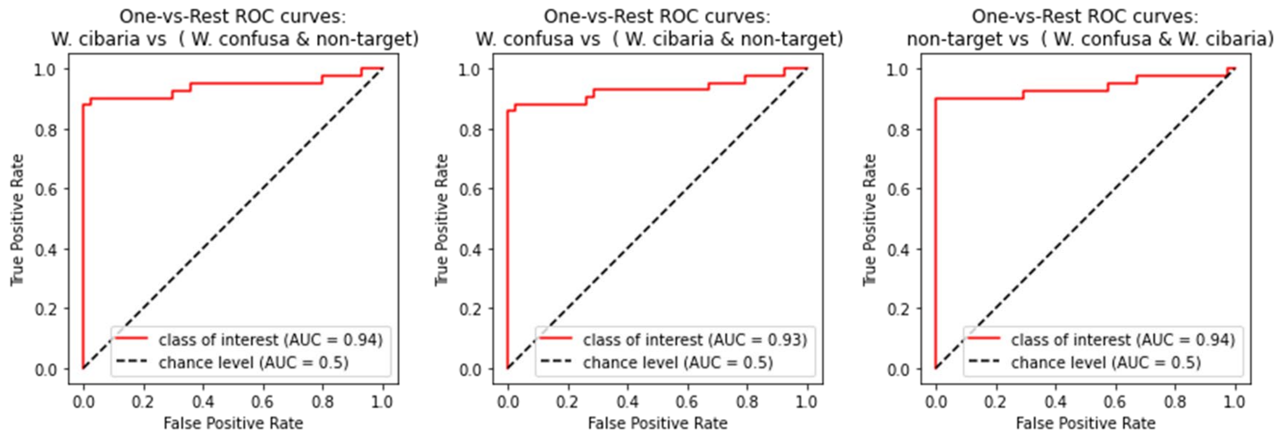

Supplementary Figure S3.

The developed PCA-KNN model was utilized to analyze the ROC curves and AU, showing the following: (Left) The classification performance between *W. cibaria* and the other two classes, (middle) The classification performance between *W. confusa* and the other two classes, (Right) The classification performance between non-target samples and the other two classes.

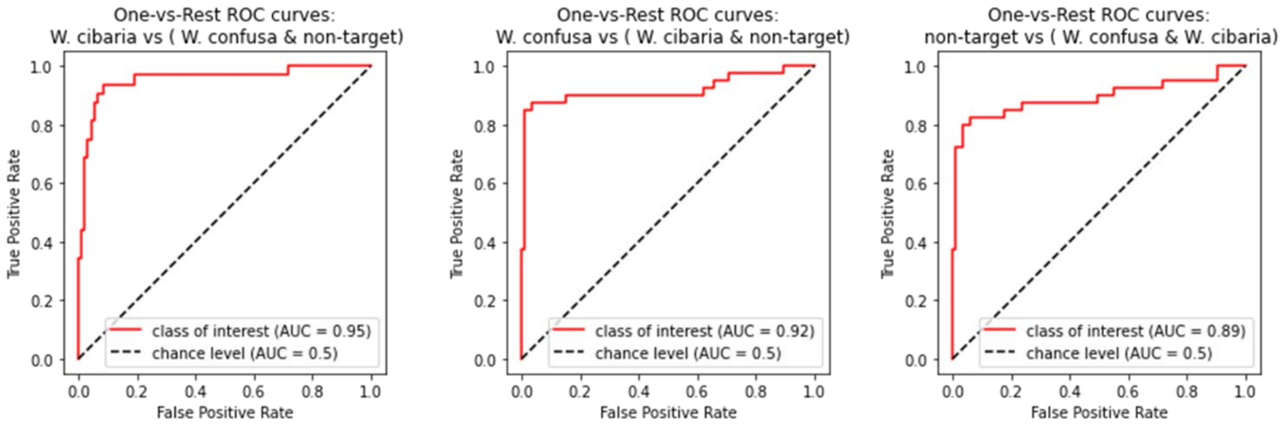

Supplementary Figure S4.

The developed SVM-sigmoid model was utilized to analyze the ROC curves and AUC, showing the following: (Left) The classification performance between *W. cibaria* and the other two classes, (middle) The classification performance between *W. confusa* and the other two classes, (Right) The classification performance between non-target samples and the other two classes.

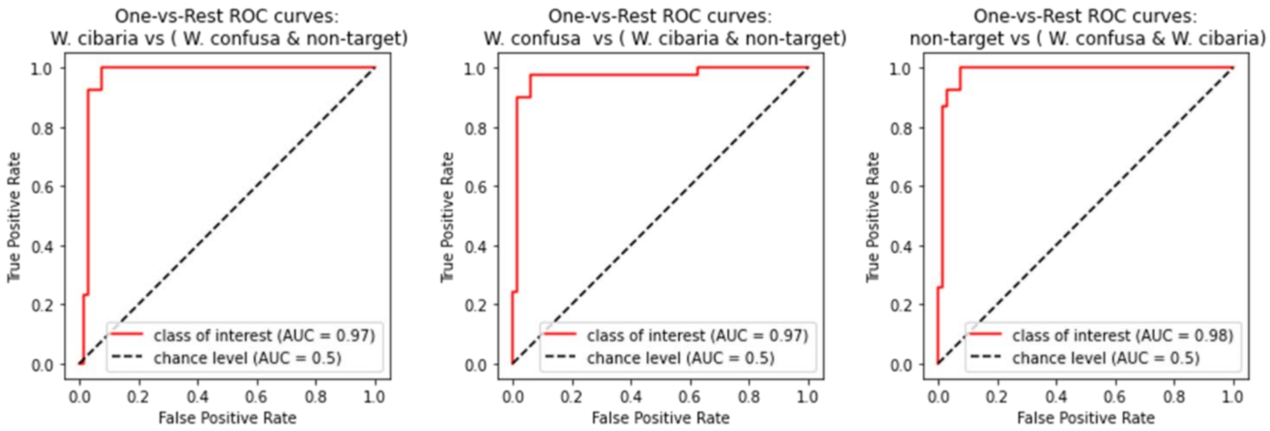

Supplementary Figure S5.

The developed Random Forest model was utilized to analyze the ROC curves and AUC, showing the following: (Left) The classification performance between *W. cibaria* and the other two classes, (middle) The classification performance between *W. confusa* and the other two classes, (Right) The classification performance between non-target samples and the other two classes.

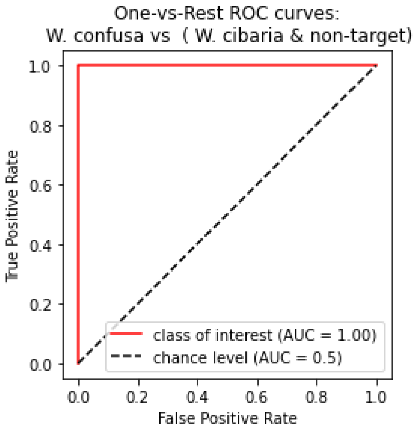

Supplementary Figure S6.

The developed Sigmoid-RBF model was employed for the analysis of ROC curves and AUC, achieving a classification accuracy of 1.0 across all three categories.

Supplementary Table S1. Identification of *W. cibaria*, *W. confusa*, and non-target strains by MALDI-TOF MS with BioTyper database and specific real-time PCR

Sample ID	Specific real-time PCR	MALDI-TOF MS with bioTyper database			
		Organism (best match)	Score Value	Organism (second-best match)	Score Value
cibaria_01	*Weissella cibaria*	*Weissella cibaria*	2.30	*Weissella cibaria*	2.11
cibaria_02	*Weissella cibaria*	*Weissella cibaria*	2.40	*Weissella cibaria*	2.18
cibaria_03	*Weissella cibaria*	*Weissella cibaria*	2.43	*Weissella cibaria*	2.22
cibaria_04	*Weissella cibaria*	*Weissella cibaria*	2.35	*Weissella cibaria*	2.11
cibaria_05	*Weissella cibaria*	*Weissella cibaria*	2.36	*Weissella cibaria*	2.15
cibaria_06	*Weissella cibaria*	*Weissella cibaria*	2.38	*Weissella cibaria*	2.21
cibaria_07	*Weissella cibaria*	*Weissella cibaria*	2.32	*Weissella cibaria*	2.27
cibaria_08	*Weissella cibaria*	*Weissella cibaria*	2.39	*Weissella cibaria*	2.25
cibaria_09	*Weissella cibaria*	*Weissella cibaria*	2.20	*Weissella cibaria*	2.18
cibaria_10	*Weissella cibaria*	*Weissella cibaria*	2.33	*Weissella cibaria*	2.13
cibaria_11	*Weissella cibaria*	*Weissella cibaria*	2.11	*Weissella cibaria*	2.01
cibaria_12	*Weissella cibaria*	*Weissella cibaria*	2.16	*Weissella cibaria*	2.13
cibaria_13	*Weissella cibaria*	*Weissella cibaria*	2.42	*Weissella cibaria*	2.11
cibaria_14	*Weissella cibaria*	*Weissella cibaria*	2.17	*Weissella cibaria*	2.12
cibaria_15	*Weissella cibaria*	*Weissella cibaria*	2.19	*Weissella cibaria*	2.15
cibaria_16	*Weissella cibaria*	*Weissella cibaria*	2.12	*Weissella cibaria*	2.07
cibaria_17	*Weissella cibaria*	*Weissella cibaria*	2.05	*Weissella cibaria*	2.05
cibaria_18	*Weissella cibaria*	*Weissella cibaria*	2.24	*Weissella cibaria*	2.08
cibaria_19	*Weissella cibaria*	*Weissella cibaria*	2.23	*Weissella cibaria*	2.23
cibaria_20	*Weissella cibaria*	*Weissella cibaria*	2.21	*Weissella cibaria*	2.14
cibaria_21	*Weissella cibaria*	*Weissella cibaria*	2.33	*Weissella cibaria*	2.31
cibaria_22	*Weissella cibaria*	*Weissella cibaria*	2.37	*Weissella cibaria*	2.35
cibaria_23	*Weissella cibaria*	*Weissella cibaria*	2.38	*Weissella cibaria*	2.35
cibaria_24	*Weissella cibaria*	*Weissella cibaria*	2.36	*Weissella cibaria*	2.25
cibaria_25	*Weissella cibaria*	*Weissella cibaria*	2.31	*Weissella cibaria*	2.22
cibaria_26	*Weissella cibaria*	*Weissella cibaria*	2.31	*Weissella cibaria*	2.24
cibaria_27	*Weissella cibaria*	*Weissella cibaria*	2.03	*Weissella cibaria*	2.01
cibaria_28	*Weissella cibaria*	*Weissella cibaria*	2.30	*Weissella cibaria*	2.29
cibaria_29	*Weissella cibaria*	*Weissella cibaria*	2.12	*Weissella cibaria*	2.12

cibaria_30	*Weissella cibaria*	*Weissella cibaria*	2.32	*Weissella cibaria*	2.09
cibaria_31	*Weissella cibaria*	*Weissella cibaria*	2.26	*Weissella cibaria*	2.19
cibaria_32	*Weissella cibaria*	*Weissella cibaria*	2.11	*Weissella cibaria*	2.09
cibaria_33	*Weissella cibaria*	*Weissella cibaria*	2.09	*Weissella cibaria*	2.07
cibaria_34	*Weissella cibaria*	*Weissella cibaria*	2.27	*Weissella cibaria*	2.26
cibaria_35	*Weissella cibaria*	*Weissella cibaria*	2.15	*Weissella cibaria*	2.03
cibaria_36	*Weissella cibaria*	*Weissella cibaria*	2.34	*Weissella cibaria*	2.24
cibaria_37	*Weissella cibaria*	*Weissella cibaria*	2.23	*Weissella cibaria*	2.20
cibaria_38	*Weissella cibaria*	*Weissella cibaria*	2.32	*Weissella cibaria*	2.22
cibaria_39	*Weissella cibaria*	*Weissella cibaria*	2.11	*Weissella cibaria*	2.00
cibaria_40	*Weissella cibaria*	*Weissella cibaria*	2.30	*Weissella cibaria*	2.13
cibaria_41	*Weissella cibaria*	*Weissella cibaria*	2.22	*Weissella cibaria*	2.10
cibaria_42	*Weissella cibaria*	*Weissella cibaria*	2.16	*Weissella cibaria*	1.99
cibaria_43	*Weissella cibaria*	*Weissella cibaria*	2.06	*Weissella cibaria*	2.01
W. cibaria KCTC 3746	*Weissella cibaria*	*Weissella cibaria*	2.16	*Weissella cibaria*	2.08
confusa_01	*Weissella confusa*	*Weissella confusa*	2.09	*Weissella cibaria*	2.08
confusa_02	*Weissella confusa*	*Weissella confusa*	2.03	*Weissella cibaria*	2.03
confusa_03	*Weissella confusa*	*Weissella cibaria*	1.91	*Weissella cibaria*	1.90
confusa_04	*Weissella confusa*	*Weissella cibaria*	1.99	*Weissella confusa*	1.85
confusa_05	*Weissella confusa*	*Weissella confusa*	2.08	*Weissella cibaria*	2.07
confusa_06	*Weissella confusa*	*Weissella cibaria*	2.12	*Weissella cibaria*	2.06
confusa_07	*Weissella confusa*	*Weissella confusa*	1.99	*Weissella confusa*	1.89
confusa_08	*Weissella confusa*	*Weissella confusa*	1.95	*Weissella confusa*	1.94
confusa_09	*Weissella confusa*	*Weissella confusa*	1.97	*Weissella cibaria*	1.92
confusa_10	*Weissella confusa*	*Weissella cibaria*	1.90	*Weissella confusa*	1.88
confusa_11	*Weissella confusa*	*Weissella confusa*	2.10	*Weissella cibaria*	2.02
confusa_12	*Weissella confusa*	*Weissella cibaria*	2.05	*Weissella confusa*	1.96
confusa_13	*Weissella confusa*	*Weissella cibaria*	2.08	*Weissella confusa*	2.04
confusa_14	*Weissella confusa*	*Weissella cibaria*	1.96	*Weissella confusa*	1.88
confusa_15	*Weissella confusa*	*Weissella cibaria*	1.89	*Weissella confusa*	1.81
confusa_16	*Weissella confusa*	*Weissella cibaria*	1.95	*Weissella confusa*	1.94
confusa_17	*Weissella confusa*	*Weissella confusa*	2.01	*Weissella cibaria*	1.87
confusa_18	*Weissella confusa*	*Weissella confusa*	1.95	*Weissella cibaria*	1.91
confusa_19	*Weissella confusa*	*Weissella cibaria*	2.02	*Weissella confusa*	1.97
confusa_20	*Weissella confusa*	*Weissella confusa*	2.07	*Weissella confusa*	1.97

confusa_21	*Weissella confusa*	*Weissella cibaria*	2.13	*Weissella confusa*	2.03
confusa_22	*Weissella confusa*	*Weissella cibaria*	2.08	*Weissella cibaria*	2.03
confusa_23	*Weissella confusa*	*Weissella cibaria*	2.08	*Weissella confusa*	2.07
confusa_24	*Weissella confusa*	*Weissella cibaria*	2.08	*Weissella confusa*	2.01
confusa_25	*Weissella confusa*	*Weissella confusa*	2.02	*Weissella confusa*	2.00
confusa_26	*Weissella confusa*	*Weissella cibaria*	2.05	*Weissella confusa*	2.01
confusa_27	*Weissella confusa*	*Weissella cibaria*	2.10	*Weissella confusa*	2.05
confusa_28	*Weissella confusa*	*Weissella cibaria*	2.04	*Weissella confusa*	1.98
confusa_29	*Weissella confusa*	*Weissella confusa*	2.06	*Weissella cibaria*	2.02
confusa_30	*Weissella confusa*	*Weissella cibaria*	2.13	*Weissella confusa*	2.12
confusa_31	*Weissella confusa*	*Weissella cibaria*	1.74	*Weissella cibaria*	1.73
confusa_32	*Weissella confusa*	*Weissella cibaria*	2.09	*Weissella confusa*	1.94
confusa_33	*Weissella confusa*	*Weissella cibaria*	2.09	*Weissella cibaria*	2.04
confusa_34	*Weissella confusa*	*Weissella cibaria*	2.05	*Weissella cibaria*	2.02
confusa_35	*Weissella confusa*	*Weissella cibaria*	2.09	*Weissella cibaria*	1.96
confusa_36	*Weissella confusa*	*Weissella cibaria*	2.13	*Weissella confusa*	2.04
confusa_37	*Weissella confusa*	*Weissella cibaria*	2.12	*Weissella cibaria*	2.03
confusa_38	*Weissella confusa*	*Weissella cibaria*	1.86	*Weissella confusa*	1.79
confusa_39	*Weissella confusa*	*Weissella cibaria*	1.79	*Weissella cibaria*	1.74
confusa_40	*Weissella confusa*	*Weissella confusa*	2.08	*Weissella cibaria*	2.00
confusa_41	*Weissella confusa*	*Weissella cibaria*	1.74	No Organism Identification Possible	1.61
confusa_42	*Weissella confusa*	*Weissella confusa*	1.74	*Weissella cibaria*	1.71
confusa_43	*Weissella confusa*	*Weissella confusa*	1.78	*Weissella confusa*	1.72
confusa_44	*Weissella confusa*	*Weissella cibaria*	1.74	*Weissella confusa*	1.72
confusa_45	*Weissella confusa*	*Weissella cibaria*	1.92	*Weissella confusa*	1.91
W. confusa KCTC 3499	*Weissella confusa*	*Weissella cibaria*	1.96	*Weissella confusa*	1.80
nontarget_01	*Weissella ceti*	No Organism Identification Possible	1.48	No Organism Identification Possible	1.35
nontarget_02	*Weissella cryptocerc*	No Organism Identification Possible	1.26	No Organism Identification Possible	1.25
nontarget_03	*Weissella diestrammenae*	*Weissella diestrammenae*	2.13	No Organism Identification Possible	1.54
nontarget_04	*Weissella halotolerans*	*Weissella halotolerans*	2.22	*Weissella halotolerans*	1.78
nontarget_05	*Weissella hellenica*	*Weissella hellenica*	2.09	No Organism Identification Possible	1.65
nontarget_06	*Weissella hellenica*	*Weissella hellenica*	1.88	No Organism Identification Possible	1.61
nontarget_07	*Weissella hellenica*	*Weissella hellenica*	1.83	No Organism Identification Possible	1.62
nontarget_08	*Weissella hellenica*	*Weissella hellenica*	1.82	No Organism Identification Possible	1.60
nontarget_09	*Weissella hellenica*	*Weissella hellenica*	1.73	No Organism Identification Possible	1.38

nontarget_10	*Weissella koreensis*	*Weissella koreensis*	2.26	No Organism Identification Possible	1.52
nontarget_11	*Weissella koreensis*	*Weissella koreensis*	2.01	No Organism Identification Possible	1.36
nontarget_12	*Weissella koreensis*	*Weissella koreensis*	2.29	No Organism Identification Possible	1.35
nontarget_13	*Weissella koreensis*	*Weissella koreensis*	2.32	No Organism Identification Possible	1.29
nontarget_14	*Weissella koreensis*	*Weissella koreensis*	2.33	No Organism Identification Possible	1.47
nontarget_15	*Weissella koreensis*	*Weissella koreensis*	2.43	No Organism Identification Possible	1.47
nontarget_16	*Weissella koreensis*	*Weissella koreensis*	2.27	No Organism Identification Possible	1.43
nontarget_17	*Weissella koreensis*	*Weissella koreensis*	2.33	No Organism Identification Possible	1.30
nontarget_18	*Weissella koreensis*	*Weissella koreensis*	2.09	No Organism Identification Possible	1.37
nontarget_19	*Weissella koreensis*	*Weissella koreensis*	2.30	No Organism Identification Possible	1.37
nontarget_20	*Weissella koreensis*	*Weissella koreensis*	2.22	No Organism Identification Possible	1.28
nontarget_21	*Weissella koreensis*	*Weissella koreensis*	2.30	No Organism Identification Possible	1.33
nontarget_22	*Weissella koreensis*	*Weissella koreensis*	1.80	No Organism Identification Possible	1.35
nontarget_23	*Weissella minor*	No Organism Identification Possible	1.58	No Organism Identification Possible	1.31
nontarget_24	*Weissella paramesenteroides*	*Weissella paramesenteroides*	1.97	*Weissella paramesenteroides*	1.74
nontarget_25	*Weissella paramesenteroides*	*Weissella paramesenteroides*	2.14	*Weissella paramesenteroides*	1.87
nontarget_26	*Weissella paramesenteroides*	*Weissella paramesenteroides*	2.05	*Weissella paramesenteroides*	1.93
nontarget_27	*Weissella paramesenteroides*	*Weissella paramesenteroides*	1.94	*Weissella paramesenteroides*	1.94
nontarget_28	*Weissella paramesenteroides*	*Weissella paramesenteroides*	2.03	*Weissella paramesenteroides*	1.94
nontarget_29	*Weissella paramesenteroides*	*Weissella paramesenteroides*	1.85	*Weissella paramesenteroides*	1.81
nontarget_30	*Weissella paramesenteroides*	*Weissella paramesenteroides*	2.03	*Weissella paramesenteroides*	1.99
nontarget_31	*Weissella paramesenteroides*	*Weissella paramesenteroides*	1.96	*Weissella paramesenteroides*	1.90
nontarget_32	*Weissella soli*	No Organism Identification Possible	1.35	No Organism Identification Possible	1.30
nontarget_33	*Weissella thailandensis*	*Weissella thailandensis*	1.90	*Weissella thailandensis*	1.77
nontarget_34	*Weissella uvarum*	*Weissella uvarum*	2.25	No Organism Identification Possible	1.57
nontarget_35	*Weissella viridescens*	*Weissella viridescens*	2.33	*Weissella viridescens*	2.13
